# Quality and continuity of information between primary care physicians and rheumatologists

**DOI:** 10.1186/s41927-019-0067-6

**Published:** 2019-05-24

**Authors:** Jenna Wong, Karen Tu, Sasha Bernatsky, Liisa Jaakkimainen, J. Carter Thorne, Vandana Ahluwalia, J. Michael Paterson, Jessica Widdifield

**Affiliations:** 1 Sunnybrook Research Institute, Holland Bone and Joint Research Program, Toronto, Ontario Canada; 2 Department of Family and Community Medicine, Institute of Health Policy, Management and Evaluation, University of Toronto, Toronto, Ontario Canada; 3 McGill University, Research Institute of the McGill University Health Centre, Montreal, Quebec, Canada; 4 Department of Family and Community Medicine, Institute of Health Policy, Management and Evaluation, ICES, University of Toronto, Toronto, Ontario Canada; 5 Southlake Regional Health Centre, Newmarket, Ontario Canada; 6 William Olser Health System, Brampton Civic Hospital, Brampton, Ontario Canada; 7 Department of Family Medicine, McMaster University, Hamilton, Ontario Canada; 8 Chronic Disease & Pharmacotherapy Program, ICES, Toronto, Ontario Canada; 9 University of Toronto Institute of Health Policy, Management and Evaluation, MG 352 – 2075 Bayview Avenue, Toronto, Ontario M4N 3M5 Canada

**Keywords:** Rheumatic diseases, Quality of care, Referral and consultation, Communication, Continuity of care, Access to care, Rheumatology

## Abstract

**Background:**

Good communication is central to a high-quality consultation process. We assessed the quality of referral information from primary care physicians (PCPs) to rheumatologists and the quality and timeliness of consultation letters from rheumatologists back to PCPs.

**Methods:**

We sampled referral letters between 2000 and 2013 from 168 PCPs and performed a retrospective chart review of 2430 patients referred to 146 rheumatologists. We assessed the completeness and timeliness of referral and consultation letters.

**Results:**

Osteoarthritis (*n* = 787, 32%) and systemic inflammatory rheumatic diseases (*n* = 745, 31%) comprised the top reasons for referral. Only 55% of referral letters summarized the patients’ medical history. Referral letters provided some details of diagnostic tests (51% labs, 34% imaging) but there was underreporting of this information on referral letters. Almost all referral letters (92%) contained details of at least one patient symptom, with the most common complaint being joint pain (54%). Only half of all referral letters provided symptom duration. The PCP only stressed an urgent consultation among 211 patients (9%). Overall, 69% of consultation letters were returned to PCPs within 30 days of consultation visit.

**Conclusion:**

We found that basic items necessary for appropriate triage, including a description of symptoms or other relevant history and results of investigations were often lacking in referral letters. The delay of receipt of consultation letters may further represent a lost opportunity for coordination and continuity of care, and may affect the quality of care patients receive.

## Background

Referral and consultation letters are generally the primary source of communication between primary care physicians (PCPs) and rheumatologists in most healthcare settings. A high-quality referral and consultation process requires good communication. Good communication between healthcare providers may reduce delays in the diagnostic care pathway, provide quality continuity of care, and improve patient and provider satisfaction [1]. Ineffective referral letters that lack pertinent information and consultation letters that are incomplete or not transferred efficiently may result in the inability to effectively prioritize patients causing delays in treatment, repetitive health services and costs, repeat prescriptions of medications that are ineffective or cause harm, and ultimately, setbacks in patient care [1, 2].

In Canada, rheumatology is one of the most frequent non-surgical specialty referrals [3]. Timely access to rheumatology care is a challenge worldwide in light of increasing workforce shortages. However, delays to rheumatology care may have significant implications for patients with systemic inflammatory conditions.

Previous studies have identified inefficiencies in written communication between PCPs and specialists and the need to improve communication between healthcare providers [4–7]. While some researchers have developed standardized forms for rheumatology referrals [5, 7, 8], at present, there is no consensus on the use of standardized forms for referral letters. As the demand for rheumatologists has outpaced supply in Canada [9, 10] and internationally [11], patients face significant delays to rheumatology care [12], which may be worsened by ineffective communication. We sought to evaluate the quality of referral information from PCPs to rheumatologists, as well as the quality and timeliness of consultation information from rheumatologists to PCPs in Ontario, Canada.

## Methods

### Study design

We performed a retrospective chart abstraction study involving patients with first-time rheumatology referrals in Ontario, Canada, which has a publicly-funded single payer healthcare system, where access to rheumatologists is dependent upon referrals. During the study period, there was no policy implementation regarding rheumatology referrals in this setting.

### Data sources

We used the Electronic Medical Record Administrative data Linked Database (EMRALD), which is comprised of electronic medical records (EMRs) from PCPs (using the same EMR software system) throughout Ontario [13]. Patient-level EMRs contain all PCP encounters, current and past medical histories, laboratory test results, prescriptions, referral letters, diagnostic tests, as well as information related to care received elsewhere and reported to the practice (such as consultation letters). Encounters with rheumatologists were verified by linking with provincial physician billing claims from the Ontario Health Insurance Plan claims history database. These data sets are linked using unique, encoded patient and physician identifiers, and are securely held and analyzed at ICES (www.ices.on.ca).

### Participants

We studied 168 PCPs, of whom 32 practiced in rural locations, 39 in suburban areas, and 97 urban areas. The mean duration of EMR use in our sample was 5 years (range 2–25). Our sample of PCPs was slightly younger with mean (range) age of 47 (28–69) years, in comparison to all Ontario PCPs [with a mean (range) age of 52 (27–79) years]. Our PCP study population also comprised more females (56% vs. 41% for all Ontario PCPs). The mean number of years in practice was 15 for our PCP participants in comparison to 19 years for all Ontario PCPs [14].

Drawing upon primary care EMRs of 268,854 patients with at least 2 years of EMR data, we identified 2430 patients with a first-time referral to a rheumatologist between 2000 and 2013.

### Data abstraction

We reviewed each patient’s record to categorize patients by their principal diagnosis or clinical impression associated with the referral. Diagnoses and clinical impressions provided by the rheumatologist where used to categorize patients. Patients were categorized into 6 main rheumatic and musculoskeletal diseases (RMDs): systemic inflammatory rheumatic diseases, osteoarthritis, regional musculoskeletal (MSK) syndromes, chronic pain, osteoporosis, and miscellaneous referrals. Systemic inflammatory rheumatic diseases were further defined. Patients where categorized according to the most serious complaint when they carried multiple RMD diagnoses.

Based on review of prior studies, we performed standardized data abstraction to ascertain the quality and completeness of referral letters, such as providing reasons for referral, relevant medical and family histories, and diagnostics tests. We also abstracted details of symptoms provided on the referral letter according to whether the symptoms were present, absent, or not reported. We also determined whether patients had diagnostic imaging performed within the 3 months preceding referral. Rheumatology consultation letters following the referral were reviewed to abstract details on whether rheumatologists were providing information related to diagnoses and general management plans.

Three trained medical abstractors performed the chart abstraction. We performed double data abstraction on an initial 10% sample of charts, whereby the data for each patient were abstracted a second time by the same abstractor and once by a different abstractor. To ensure good agreement, we required κ scores for inter- and intra-rater reliability to exceed 0.85 before commencing full data abstraction. For all patients, an independent abstractor (J.W.) also performed double data abstraction related to assigning patients to diagnostic categories.

### Statistical analysis

Descriptive statistics were used to describe patients and the contents of the letters, stratified according to diagnostic category. We assessed the frequency of general details provided on referral letters (patient history and laboratory results), details of symptoms provided on the referral letter, actual diagnostic imaging performed on the patient in contrast to what was reported on the referral letter, and details and timeliness of consultation letters.

Analyses were performed on coded data using SAS, version 9.2, and Microsoft SQL Server 2012.

## Results

Among 2430 patients referred to a rheumatologist, 1682 (69.2%) were female, with a mean (standard deviation) age of 53.0 (16.3) years at time of referral. Most referrals occurred between 2005 and 2013. Osteoarthritis (*n* = 787, 32%), systemic inflammatory rheumatic diseases (*n* = 745, 31%), and regional musculoskeletal conditions (*n* = 395, 16%) comprised the top reasons for referral.

Most referral letters (98.9%) stated a general reason for the referral (Table 1). The PCPs requested urgent consultations for 211 patients (8.7%); most frequently for RA patients (21.8%). Only 55.2% of all referral letters summarized the patients’ medical history, 56.9% described medication history, 16.1% family history, and 12.8% employment history. Overall, 1245 (51.2%) referral letters contained details of relevant laboratory test results. Laboratory tests were most frequently reported for patients with systemic inflammatory conditions (66.0%), and for miscellaneous referrals (67.9%) – including asymptomatic patients with abnormal results but no clinical diagnosis, Table 1.

**Table 1:** General Details Provided by PCPs on Referral Letters, %

Diagnosis	Reason for referral	Urgent Consult Needed	Medical history	Family history	Medication history	Employment history	Relevant Laboratory Results
All patients *n* = 2430	98.9	8.7	55.2	16.1	56.9	12.8	51.2
Systemic inflammatory *n* = 745	99.2	14.0	56.6	18.7	62.8	12.8	66.0
RA *n* = 120	99.2	20.8	52.5	20.0	70.8	13.3	76.7
IA, other *n* = 167	100.0	13.8	53.9	19.2	63.5	18.0	72.5
Crystal *n* = 122	100.0	13.1	64.8	16.4	76.2	11.5	50.0
PMR *n* = 66	100.0	12.1	56.1	NR	65.2	NR	75.8
SpA *n* = 76	97.4	11.8	51.3	17.1	46.1	17.1	52.6
PsA *n* = 44	93.2	NR	63.6	13.6	50.0	13.6	56.8
Other SARDs^b^ *n* = 150	100.0	12.7	57.3	26.0	56.0	8.0	68.7
Osteoarthritis *n* = 787	98.4	5.2	48.4	15.4	53.8	13.5	45.1
Regional MSK syndromes *n* = 395	98.7	4.6	51.7	10.4	49.9	11.4	30.9
Chronic pain conditions *n* = 346	99.4	9.5	52.3	17.9	54.1	15.9	55.2
Osteoporosis/osteopenia *n* = 45	100.0	NR	73.3	13.3	80.0	NR	20.0
Other/miscellaneous^a^ *n* = 112	98.2	10.7	67.0	20.5	64.3	7.1	67.9

*Abbreviations*: *RA* rheumatoid arthritis, *IA* inflammatory arthritis, *NR* not reportable (to protect patient privacy), *PMR* polymyalgia rheumatica, *SpA* spondyloarthritis, *PsA* psoriatic arthritis, *SARDs* systemic autoimmune rheumatic diseases, *MSK* musculoskeletal

^a^Miscellaneous referrals such as abnormal tests

Values are the percentage with the denominator being the N within each diagnosis category

^b^SARDs include lupus, vasculitis, scleroderma, Sjögren’s syndrome, dermatomyositis, polymyositis, Raynaud’s phenomenon, sarcoidosis, etc. (not defined in the previous categories)

In total 820 (33.7%) referral letters mentioned relevant diagnostic imaging, which were most frequently reported for patients with osteoarthritis (46.4%). However, there was a higher proportion of diagnostic imaging performed on patients within the 3 months preceding referral across all diagnostic categories than compared to what was conveyed in the referral letters (Table 2).

**Table 2:** Diagnostic Imaging Reported on Referral Letters Versus Actual Imaging Performed on the Patient, %

Diagnosis	Any Diagnostic Imaging Reported on the Referral Letter	Actual Imaging Performed on the Patient^a^		
		Radiographs	Ultrasound	MRI
All patients *n* = 2430	33.7	44.7	15.2	6.0
Systemic inflammatory *n* = 745	30.0	49.4	14.8	6.3
RA *n* = 120	32.5	56.7	10.0	5.0
IA, other *n* = 167	32.3	52.1	16.8	5.4
Crystal *n* = 122	40.1	54.9	8.2	NR
PMR *n* = 66	13.6	43.9	27.3	NR
SpA *n* = 76	40.8	50.0	13.2	11.8
PsA *n* = 44	36.4	45.5	13.6	NR
Other SARDs *n* = 150	12.0	39.3	17.3	8.0
Osteoarthritis *n* = 787	46.4	49.9	14.1	5.5
Regional MSK syndromes *n* = 395	34.9	41.5	17.7	5.8
Chronic pain conditions *n* = 346	22.0	33.0	15.9	7.2
Osteoporosis/osteopenia *n* = 45	24.4	37.8	NR	NR
Other/miscellaneous^b^ *n* = 112	12.5	27.7	17.0	6.3

Values are the percentage with the denominator being the N within each diagnosis category

*Abbreviations*: *RA* rheumatoid arthritis, *IA* inflammatory arthritis, *PMR* polymyalgia rheumatica, *SpA* spondyloarthritis, *PsA* psoriatic arthritis, *SARDs* systemic autoimmune rheumatic diseases, *MSK* musculoskeletal

*NR* Not reportable (to protect patient privacy)

^a^Diagnostic imaging performed within the 3 months period prior to referral

SARDs include lupus, scleroderma, vasculitis, Sjögren’s syndrome, dermatomyositis, polymyositis, Raynaud’s phenomenon, sarcoidosis, etc. (not defined in the previous categories)

^b^Miscellaneous referrals such as abnormal tests

Almost all referral letters (92.2%) contained details of at least one symptom (Table 3), with the most common being joint pain (51.1%). In total, 64.7% of referral letters indicated the anatomical site (or distribution of joints) affected associated with the reason for referral. Half of all referral letters provided an indication of the duration of symptoms (51.1%). There was variation in the types of symptoms reported (whether present or absent in the patient) across diagnostic categories. PCPs were most likely to report on joint pain for osteoarthritis patients (63.5%) in referral letters compared to other conditions.

**Table 3:** Details of Symptoms Provided (whether present or absent for the patient) on the Referral Letter

	Overall *n* = 2430	Systemic inflammatory *n* = 745	Osteoarthritis *n* = 787	Regional MSK *n* = 395	Chronic Pain *n* = 346	Osteoporosis/Osteopenia *n* = 45	Misc.^a^ *n* = 112
At least 1 symptom mentioned	92.2%	92.6%	95.6%	93.4%	92.8%	33.3%	83.9%
Anatomical Site(s)	64.7%	66.2%	75.3%	65.8%	52.6%	13.3%	33.0%
Joint Pain	51.1%	52.5%	63.5%	50.9%	34.7%	NR	25.0%
Symptom/disease duration provided	50.3%	55.4%	48.0%	49.1%	54.9%	15.6%	35.7%
Swollen joints	22.6%	34.9%	22.1%	16.7%	10.7%	NR	9.8%
Generalized pain	16.6%	15.6%	8.8%	12.7%	45.7%	NR	9.8%
“Arthritis”	15.0%	14.5%	26.6%	6.3%	4.9%	NR	NR
Morning stiffness	9.3%	12.2%	9.7%	6.6%	8.7%	NR	NR
Joint redness	5.1%	7.5%	4.6%	4.8%	2.6%	NR	NR
Impaired abilities with daily living	7.5%	6.2%	8.8%	7.6%	9.8%	NR	NR
Fatigue	4.7%	4.4%	1.9%	NR	16.8%	NR	NR
Deformed joints	2.6%	2.6%	4.3%	2.0%	NR	NR	NR
Impaired sleep	2.0%	0.8%	2.3%	2.0%	4.9%	NR	NR

Values are the percentage with the denominator being the N within each diagnosis category. Systemic Inflammatory conditions include rheumatoid arthritis, inflammatory arthritis, polymyalgia rheumatica, spondyloarthritis, psoriatic arthritis, gout and other crystal arthropathies, other SARDs (lupus, scleroderma, vasculitis, Sjögren’s syndrome, dermatomyositis, polymyositis, Raynaud’s phenomenon, sarcoidosis, etc); *MSK* musculoskeletal, *Misc* miscellaneous

*NR* Not reportable (to protect patient privacy)

^a^Miscellaneous referrals such as abnormal tests

For patients with systemic inflammatory conditions, RA patients had the most symptoms (to be present in these individuals) documented on the referral letters (Table 4). Yet, only 52.5% of RA patients had symptom duration explicitly reported, only 49.2% had mention of swollen joints and 64.2% had mention of tender joints. Across diagnostic categories, symptoms impairing patients’ function (such as fatigue, morning stiffness, or impaired abilities with daily living) were infrequently reported.

**Table 4:** Symptoms Reported on the Referral Letter to be Present in Patients with Systemic Inflammatory Conditions

	RA *n* = 120	IA, other *n* = 167	Crystal *n* = 122	PMR *n* = 66	SpA *n* = 76	PsA *n* = 44	Other SARDs^a^ *n* = 150
Anatomical Site(s)	71.7%	71.3%	68.0%	31.8%	57.9%	77.3%	36.0%
Joint Pain	64.2%	68.9%	56.6%	24.2%	60.5%	56.8%	24.0%
Symptom duration	52.5%	44.3%	31.1%	39.4%	32.9%	38.6%	36.7%
Swollen joints	49.2%	42.5%	39.3%	NR	15.8%	52.3%	16.7%
“Arthritis”	26.7%	15.6%	16.4%	NR	9.2%	22.7%	4.0%
Morning stiffness	18.3%	15.0%	NR	12.1%	18.4%	15.9%	4.0%
Impaired abilities with daily living	8.3%	4.8%	6.6%	10.6%	NR	NR	4.7%
Generalized pain	6.7%	15.6%	NR	48.5%	14.5%	NR	18.7%
Deformed joints	5.8%	NR	NR	NR	NR	NR	NR
Rash(es)	NR	4.2%	NR	NR	NR	NR	20.7%
Raynaud’s	NR	NR	NR	NR	NR	NR	18.7%
Fatigue	NR	3.6%	NR	NR	NR	NR	10.0%

Values are the percentage with the denominator being the N within each diagnosis category. *RA* rheumatoid arthritis, *IA* inflammatory arthritis, *PMR* polymyalgia rheumatica, *SpA* spondyloarthritis, *PsA* psoriatic arthritis

*NR* Not reported due to small cell size

^a^Other SARDs include systemic lupus, vasculitis, scleroderma, Sjögren’s syndrome, dermatomyositis, polymyositis, Raynaud’s phenomenon, sarcoidosis, etc. (not defined in the previous categories)

Among 2430 referrals, 2015 (82.9%) patients were subsequently seen by 146 rheumatologists (according to rheumatology claims data). Among these patients, 1899 (94.2%) patients had rheumatology consultation letters present in the PCP records and 68.8% were returned within 30 days of consultation. Most consultation letters (93.1%) provided a diagnosis or clinical impression, 91.5% provided a follow-up plan, 84.0% specified the care provider responsible for follow-up, 52.2% detailed instructions provided to the patient, and 17.3% mentioned or recommended allied health care providers to be involved in the patient’s care, Table 5. In our sample, 17% if referrals did not result in a rheumatology consultation, 68 (1.6%) patients had evidence that they subsequently cancelled or missed their consultation appointment and only 87 patients (2.1%) had explicit documentation of a rheumatologist declining to see the patient (the majority were for non-systemic inflammatory conditions). Among these 87 declined referrals, the main reasons provided by rheumatologists for declined referrals were that they did not provide consultations specific to the diagnosis (26.4%), or that a consultation was not required (24.1%). Among 19 (21.8%) of declined referrals, no reason was provided.

**Table 5:** Details Provided on Consultation Letters among patients Seen by 146 Rheumatologists and Timeliness of Return to PCPs, %

	Provisional diagnosis/clinical impression	General follow-up	Provider responsible for follow-up	Instructions that were given to patients	Allied healthcare providers involved in care	Consultation Letter Returned within 1 month	Consultation Letter Returned within 3 months	Consultation Letter Returned within 1 year
Overall	93.1	91.5	84.0	52.2	17.3	68.8	78.9	83.1
Systemic Inflammatory Conditions	95.0	93.4	90.5	56.9	10.2	69.2	81.0	87.4
Osteoarthritis	93.5	91.7	79.9	51.3	24.6	71.0	79.0	80.9
Regional MSK syndromes	93.4	91.5	83.3	50.2	22.0	69.4	65.6	83.3
Chronic pain conditions	86.5	84.9	77.1	48.2	18.4	59.4	72.3	77.9
Osteoporosis/osteopenia	97.0	100.0	87.9	60.6	NR	75.0	82.1	82.1
Other/miscellaneous^a^	92.0	90.7	86.7	37.3	NR	74.4	82.9	82.9

Values are the percentage

*NR* Not reported due to small cell size

^a^Miscellaneous referrals such as abnormal tests

## Discussion

This study presents data on the current state of rheumatology referral by PCPs and rheumatologist consultation information in Ontario, Canada. We found that basic items necessary for appropriate triage, including a description of symptoms or other relevant history and results of investigations, were often lacking in referral letters. Consultation letters from rheumatologists were reasonably complete, however approximately one third of consultation letters were not returned to the PCP within 30 days of seeing the patient.

Our study, which sampled from a large population of PCPs, reinforces findings from previous Canadian and international reports from single rheumatology centers that referral letters lack potentially important details [7, 15–20]. In our study, 45% of referral letters lacked a summary of the patient’s medical history. Perhaps PCPs using EMRs infrequently summarize medical histories as this information is often summarized in the cumulative patient profile portion of the EMR software system, which can be printed separately and transferred along with referral letters. Conversely, PCPs may also not fully use their EMRs efficiently to transfer this information directly onto referral letters. For PCPs using an EMR system, efficiencies may be gained in the ability to transfer patient records between providers (either complete medical histories or patient summaries). On the other hand, these efficiencies may be lost when PCPs fail to summarize pertinent information within referral letters and rheumatologists are required to extensively review more detailed medical files in order to identify relevant information. Moreover, opportunities may be lost by PCPs exploiting this practice as the quality of referral letters are generally contingent on the knowledge of the PCP [4]. Understanding the content of what is required to inform a high quality referral letter can create opportunities to improve on diagnostic skills, knowledge pertaining to RMDs, and strengthening care partnerships with specialists.

The presence of comprehensive and easily accessible information in referrals letters is likely to impact on the decision-making process for patient appointments, regardless of the type of health care system. In many countries, a traditional PCP-to-rheumatologist referral process occurs in which new patients are referred directly to a specific rheumatologist [21]. We were unable to assess the quality of referral information on timeliness of care, as it was not clear what additional information was transferred along with referral letters (e.g. complete patient record or recent investigations) or whether rheumatology practices were requesting more information on individual patients. However, patients with RA and inflammatory arthritis contained more symptoms reported on referral letters (Table 4). Previously, we have shown that these individuals have shorter wait times to rheumatology (median of 66 and 55 days, respectively) compared to other RMDs [12].

Indeed, the majority of rheumatologists recently surveyed in another Canadian setting were not satisfied with the quality and completeness of referral letters, making it difficult to triage patients appropriately, resulting in requests for more information (such as laboratory test results) and contributing to delays [8].

We believe our study highlights that communication and continuity of information between PCPs and rheumatologists could be improved. Future efforts to improve the referral process could ultimately improve both physician satisfaction and quality of patient care. Local continuing medical education activities at the primary care level would likely be inefficient at a global scale. The use of standardized referral templates has demonstrated to be successful [5, 7, 8, 22–25] but universal adoption has been problematic. It is clear that rheumatology needs a coherent strategy to improve the referral process, such as requiring referring PCPs to use a validated general rheumatology referral form [26].

We acknowledge some potential limitations of our study. As a retrospective study, all data and conclusions are dependent on the accuracy of documentation and coding of the medical records and referral letters. This raises the possibility of misclassification between diagnostic categories. We were unable to assess the agreement of clinical diagnoses between rheumatologists and PCPs, as a suspected clinical diagnosis was rarely indicated on referral letters. In general, patients with more classic disease presentation or more active disease may be more likely to have suspected diagnoses within referral letters and the retrospective nature of our study would over estimate the agreement. Furthermore, we did not scrutinize the quality of consultation letters as thoroughly as the contents of referral letters. In the context of rheumatology, there is lack of consensus on what defines a high quality consultation letter and we only assessed some general components of what is recommended to be included in general consultation letters [27].

## Conclusions

In summary, we identified an under reporting of key information within rheumatology referral letters and a delay of receipt of consultation letters, which may represent a lost opportunity for coordination and continuity of rheumatology care, and may ultimately affect the quality of care. Engagement with PCPs and rheumatologists to identify feasible and optimal ways to improve communication is needed.

## Abbreviations

EMRALD: Electronic Medical Record Administrative data Linked Database
EMRs: Electronic medical records
IA: Inflammatory arthritis
PCPs: Primary care physicians
PMR: Polymyalgia rheumatica
PsA: Psoriatic arthritis
RA: Rheumatoid arthritis
RMDs: Rheumatic and musculoskeletal diseases
SARDs: Systemic autoimmune rheumatic diseases
SpA: Spondyloarthropathies
